# Digitalizing Clinical Guidelines: Experiences in the Development of Clinical Decision Support Algorithms for Management of Childhood Illness in Resource-Constrained Settings

**DOI:** 10.9745/GHSP-D-22-00439

**Published:** 2023-08-28

**Authors:** Fenella Beynon, Frédérique Guérin, Riccardo Lampariello, Torsten Schmitz, Rainer Tan, Natschja Ratanaprayul, Tigest Tamrat, Karell G. Pellé, Gaud Catho, Kristina Keitel, Irene Masanja, Clotilde Rambaud-Althaus

**Affiliations:** a Swiss Tropical and Public Health Institute, Basel, Switzerland.; b University of Basel, Basel, Switzerland.; c Geneva Science-Policy Interface, University of Geneva, Switzerland.; d Terre des Hommes, Lausanne, Switzerland.; e D-tree, USA.; f Digital and Global Health Unit, Unisanté, Center for Primary Care and Public Health, Lausanne, Switzerland.; g Ifakara Health Institute, Dar es Salaam, Tanzania.; h Department of Digital Health and Innovations, World Health Organization, Geneva, Switzerland.; i UNDP/UNFPA/UNICEF/World Bank Special Program of Research, Development and Research Training in Human Reproduction (HRP), Department of Sexual and Reproductive Health and Research, World Health Organization, Geneva, Switzerland.; j FIND, Geneva, Switzerland.; k Division of Infectious Diseases, Geneva University Hospital and Faculty of Medicine, University of Geneva, Geneva, Switzerland.; l Global Health Institute, University of Geneva, Geneva, Switzerland.; m Department of Pediatric Emergency Medicine, Department of Pediatrics, Inselspital, University Hospital Bern, University of Bern, Bern, Switzerland.; n Médecins Sans Frontières Switzerland, Operational Center Geneva, Geneva, Switzerland.

## Abstract

Clinical decision support systems (CDSSs) can strengthen the quality of integrated management of childhood illness (IMCI) in resource-constrained settings. Several IMCI-related CDSSs have been developed and implemented in recent years. Yet, despite having a shared starting point, the IMCI-related CDSSs are markedly varied due to the need for interpretation when translating narrative guidelines into decision logic combined with considerations of context and design choices. Between October 2019 and April 2021, we conducted a comparative analysis of 4 IMCI-related CDSSs. The extent of adaptations to IMCI varied, but common themes emerged. Scope was extended to cover a broader range of conditions. Content was added or modified to enhance precision, align with new evidence, and support rational resource use. Structure was modified to increase efficiency, improve usability, and prioritize care for severely ill children. The multistakeholder development processes involved syntheses of recommendations from existing guidelines and literature; creation and validation of clinical algorithms; and iterative development, implementation, and evaluation. The common themes surrounding adaptations of IMCI guidance highlight the complexities of digitalizing evidence-based recommendations and reinforce the rationale for leveraging standards for CDSS development, such as the World Health Organization’s SMART Guidelines. Implementation through multistakeholder dialogue is critical to ensure CDSSs can effectively and equitably improve quality of care for children in resource-constrained settings.

## Abstract (precis)

Clinical decision support systems can strengthen the quality of IMCI but can vary because of the need for interpretation when translating narrative guidelines into decision logic combined with considerations of context and design choices.

## Key Messages

- Clinical decision support systems (CDSSs)—digital tools that support health care providers to improve quality of care—have been developed by various groups to support the management of childhood illness in low- and middle-income countries.
- CDSSs can differ substantially because of the need for interpretation when translating narrative guidelines into decision logic.
- Relative to paper-based integrated management of childhood illness (IMCI) guidance, 4 CDSS developers all made adaptations to scope, content, and structure to cover a broader range of conditions, enhance precision, support rationale resource use, expedite care for severely ill children, and improve usability and acceptability.
- The extent of adaptations highlights the need for guideline developers to provide greater precision in their recommendations to reduce the potential for divergence from evidence-based practice during digitalization.
- Multistakeholder efforts are needed to build and adhere to standards for CDSS development to ensure transparency and accountability and to maximize impact on health and quality-of-care outcomes.

## INTRODUCTION

Poor quality of care results in an estimated 5 million excess deaths annually in low- and middle-income countries.^1^ Emphasizing the need to improve quality of care for children to reduce mortality in children aged younger than 5 years,^2^ the World Health Organization (WHO) Integrated Management of Childhood Illness (IMCI) strategy includes simple, structured guidance to support health care providers in implementing evidence-based recommendations.^3^^–^^6^ Focused on common conditions contributing to the highest burden of morbidity and mortality, the IMCI strategy may reduce child mortality by 15% when fully implemented.^3^^,^^6^ Since its launch in the 1990s, the IMCI strategy has been rolled out to more than 100 countries.^3^^,^^4^ But adherence by health care providers, faced with the difficult task of providing care with limited training and resources, remains low in many countries.^7^^,^^8^

Clinical decision support systems (CDSSs)—digital tools that provide tailored guidance based on individual patient information—have been recommended by WHO to support health care providers’ adherence to guidelines.^9^^–^^11^ However, substantial heterogeneity in acceptability, uptake, and impact has been found, attributable to a complex interplay of differences in context, design, and implementation.^12^^–^^14^

CDSSs provide tailored guidance to support health care providers’ adherence to guidelines; however, substantial heterogeneity in acceptability, uptake, and impact has been found.

CDSS developers, working with implementers and users, are confronted by a multitude of design choices, including which clinical recommendations to incorporate, how to translate narrative guidance into decision logic, and what standards and technology to use to implement them.^15^^–^^17^ Differences in approach to and documentation of these decisions have resulted in wide variation in CDSS quality and transparency.^18^^,^^19^ Multisectoral experts have highlighted the particular importance of transparency of the clinical algorithms used in CDSSs to ensure stakeholders are able to understand if (and how) the decision logic reflects or differs from evidence-based recommendations in guidelines and peer-reviewed literature.^20^

In recognition of these challenges, WHO recently launched the SMART (Standards-based, Machine-readable, Adaptive, Requirements-based, and Testable) Guidelines initiative. The SMART Guidelines provide a standardized approach to the digitalization of WHO recommendations, with the aim of facilitating more rapid and effective uptake of evidence-based practice.^19^ Elaborating on earlier work by Boxwala,^16^ the WHO SMART Guideline framework is organized in different “knowledge layers” (Box 1).^19^^,^^21^ Since 2021, digital adaptation kits (Layer 2) have been published for antenatal care, family planning, and HIV, with forthcoming kits planned for immunization and child health in humanitarian settings.^22^

BOX 1 The 5 Knowledge Layers of the World Health Organization SMART Guideline Framework^19^^,^^21^

1. **Narrative**: guideline and data recommendations
2. **Operational**: digital adaptation kits, comprised of semistructured documentation of operational and functional requirements
3. **Machine readable**: structured, software-neutral specifications, code, terminology, and interoperability standards
4. **Executable**: reference software, able to execute static algorithms and interoperable digital components and deliver operational and functional requirements
5. **Dynamic**: executable dynamic algorithms that are trained and optimized with advanced analytics to achieve prioritized outcomes

Before this move toward standardization by WHO, several CDSSs aimed at supporting health care providers in the management of childhood illness in primary care in low- and middle-income countries had emerged, with demonstrated improvements in quality of care, health outcomes, and antimicrobial stewardship.^23^^–^^29^ Having been developed by various groups and for a wide range of contexts, the resulting clinical algorithms (Layer 2) differ despite a shared starting point.

In this technical note, we share lessons learned by the eIMCI working group—a collaboration of IMCI-related CDSS practitioners, scientists, and policy actors—and highlight considerations when developing clinical algorithms (i.e., from Layer 1 to Layer 2). Though not representative of all IMCI-related CDSSs, the group—formed through Geneva Health Forum CDSS sessions—includes representatives from organizations involved in the development of clinical algorithms for 4 major child health CDSSs implemented in collaboration with ministries of health, nongovernmental organizations, academic institutes, and other stakeholders in 15 countries.

Through a series of 10 formal working group meetings, informal discussions, and written exchanges via semistructured questionnaires and work on shared documents between October 2019 and April 2021, we compared the clinical algorithms of these 4 CDSSs with each other and with paper-based IMCI guidance. The comparison was structured around the following axes, defined iteratively over the course of group discussions: (1) context and objectives of the interventions CDSS support, (2) adaptations relative to the WHO IMCI chart booklet,^5^ and (3) methods and processes of CDSS development.

## CDSS OBJECTIVES AND CONTEXTS OF DEVELOPMENT AND IMPLEMENTATION

The CDSSs we compared are all knowledge-based systems (i.e., rule-based, rather than “non-knowledge-based,” which can extract rules using machine learning), given our focus on translation from clinical recommendations to decision logic. They all provide step-by-step guidance for consultations for sick children in facility-based primary care in resource-constrained settings to enable comparability (rather than those developed for only a portion of the consultation such as medication dosing, for well children, or for use at the community level based on integrated community case management).^30^

The 4 CDSSs, detailed in Table 1, all aim to support health care providers to manage sick children in primary care to contribute to reducing morbidity and mortality in children aged younger than 5 years and improving the rational use of resources.^23^^–^^25^^,^^28^^,^^31^^–^^42^ The developing and implementing organizations also acknowledged that they aimed to leverage CDSS potential to: (1) be updated more readily than paper-based guidelines; (2) contribute to health worker development of knowledge and skills through on-the-job training; and (3) enhance the quality and accessibility of data for decision-making and feedback to health care providers.

**TABLE 1.** Clinical Decision Support Systems for Pediatric Primary Care in Resource-Constrained Settings Selected for Comparative Assessment

**CDSS and Software; Platform**	**Developing Agency and Collaborators**	**Intervention Type**	**Countries, Dates Implemented, and Implementation Scale**
IeDA; Commcare/Dimagi	Terre des hommes MOHs, LSHTM, Centre Muraz, UNIGE, FIND, WHO, Tableau, Cloudera, ITU, IPE Global	QI/HSS/Research	Since 2010, >20 million consultations; Burkina Faso, 2010, 1844 HF Mali, 2017, 50 HF Niger, 2019, 2 HF India, 2020, 296 HF Guinea, 2022, 15 HF
ALMANACH; Commcare/Dimagi	SwissTPH and IHI MOHs, ICRC, Adamawa SPHCDA, Somali Red Crescent Society	QI in humanitarian settings/HSS; Tanzania: Research pilot	Since 2015, >535,000 consultations; Tanzania, 2011–2012, 6 HF (research) Afghanistan, 2015–2017, 3 HF Nigeria, 2016–ongoing, 412 HF Somalia, 2020–ongoing, 23–28 HF Libya, 2022–ongoing, 6–28 HF
MSFeCARE-Ped; Logiak/Things Prime	MSF Switzerland MOHs	QI in humanitarian settings	MSF projects 2017–ongoing in 16 projects, 39 HF, >410,000 consultations: Central African Republic Democratic Republic of Congo Kenya Mali Mozambique Niger South Sudan (historically Chad, Nigeria, and Tanzania)
ePOCT; Mangologic	SwissTPH IHI, MOH	Research/Pilot	Tanzania 2014–2016; 9 HF; 1,586 consultations
ePOCT+; medAL-creator and medAL-reader/Wavemind	SwissTPH and Unisanté MOHs, PATH, IHI, KGMU, UCAD, UON, IHI, NIMR-MMRC, MOHs, EPFL, FIND	QI/HSS/Research	2019–ongoing; >310,000 consultations in large-scale research studies: TIMCI project in India, 9 HF pilot Kenya, 60 HF Senegal, 60 HF Tanzania, 24 HFDynamic project in Tanzania, 60 HF Rwanda, 39 HF

Abbreviations: CDSS, clinical decision support system; EPFL, École polytechnique fédérale de Lausanne; ePOCT, electronic point-of-care test; FIND, the global alliance for diagnostics; HF, health facilities; HSS, health systems strengthening; ICRC, International Committee of the Red Cross; IHI, Ifakara Health Institute; ITU, International Telecommunication Union; KGMU, King George’s Medical University, Lucknow; LSHTM, London School of Hygiene and Tropical Medicine; MOH, ministry of health; MSF, Médecins Sans Frontières; NIMR-MMRC, National Institute of Medical Research Mbeya Medical Research Centre; QI, quality (of care) improvement; RCT, randomized controlled trial; SDC, Swiss Agency for Development and Cooperation; SPHCDA, State Primary Health Care Development Agency; SwissTPH, Swiss Tropical and Public Health Institute; TIMCI, Tools for Integrated Management of Childhood Illness; UCAD, Université Cheikh Anta Diop de Dakar; UON, University of Nairobi; UNIGE, University of Geneva; WHO, World Health Organization.

Although these tools were developed with shared aims, they were developed and adapted for different contexts. The organizations that initiated their development have taken different intervention approaches and reached different scales in field implementation (Table 1).

## IDENTIFIED ADAPTATIONS FROM THE IMCI CHART BOOKLET

Differences between the clinical algorithms are to be expected, given their differing implementation contexts, in line with the IMCI strategy of adapting generic global guidelines to local epidemiology and available resources. Yet, above and beyond contextual differences, 3 major categories of adaptation—scope, content, and structure—were identified as common to all the CDSSs when compared to paper-based IMCI guidance. The extent of these adaptations differed according to the individual CDSS.

### Extended Scope

The WHO IMCI chart booklet proposes a simple syndromic approach.^5^ For children aged 2–59 months, this is based on the clinical assessment of a few basic danger signs: assessment of 4 main symptom groups; (1) cough/difficulty breathing, (2) diarrhea, (3) fever (predominantly for malaria and measles), (4) ear problems; and screening for malnutrition, anemia, and HIV. This narrow scope reflected a desire to strike a balance between ensuring low-skilled health care providers were equipped with the guidance to appropriately identify and manage the main causes of morbidity and mortality in primary care while not feeling overburdened with overly complex guidelines.

Relative to IMCI, the scope of most of the clinical algorithms compared was extended to cover a broader range of clinical conditions, such as dermatological or throat problems (Table 2). One tool extended to include over 50 additional diagnoses and a wider age range coverage.

**TABLE 2.** Comparison of Scope of Clinical Decision Support Systems to IMCI Guidelines

		**IMCI**	**IeDA**	**ALMANACH**	**MSF-eCARE**	**ePOCT+**
Age	0–2 months	X	X	Planned	Planned	X
	2–59 months	X	X	X	X	X
	5–14 years					Some countries
Main symptoms	Danger signs	X	X	X	X	X
	Cough/difficulty breathing	X	X	X	X	X
	Diarrhea	X	X	X	X	X
	Fever (malaria, measles)	X	X	X	X	X
	Ear problem	X	X	X	X	X
	Skin problem	HIV-related	HIV-related	X	X	X
	Fever (non-malaria)	Mentioned	Mentioned	X	X	X
	Sore throat			X	X	Some countries
	Mouth problem				X	Some countries
	Eye problem				X	Some countries
	Abdominal pain				X	Some countries
	Trauma					Some countries
Systematic screening and management	Anemia	X	X	X	X	X
	Malnutrition	X	X	X	X	X
	HIV	X	X	X	X	Some countries
	TB		Pilot			X
Prevention	Vitamin A	X	X	X		X
	Deworming	X	X	X		X
	Vaccination	X	X	X	X	X
	Feeding	X	X	X		X
Point-of-care and lab tests	Malaria	X	X	X	X	X
	HIV	X		X	X	Some countries
	Hemoglobin			X	Optional	Some countries
	Urine			X	X	Some countries
	Pulse oximetry		Pilot		Optional	X
	Glucose			X	Optional	Some countries
	Stool microscopy			X		Some countries
	Strep-A			X		
	Dengue		Pilot			Some countries
	Typhoid			X		Some countries
	C-reactive protein					Some countries

Abbreviations: ALMANACH, Algorithm for the Management of Childhood Illnesses; ePOCT+, Electronic Point-of-Care Test Plus; IeDA, Integrated eDiagnosis Approach; IMCI, Integrated Management of Childhood Illness.

The rationale for extending the scope was to support health care providers to implement evidence-based practice for a wider range of problems, thus enhancing quality of care for children presenting with non-IMCI conditions. Additionally, some organizations noted that broadening scope encouraged adoption by health care providers who found the tools more relevant to their practice.

### Content

All tools also included additional or modified content, such as new diagnostic tests or changes to diagnostic criteria, to enhance sensitivity and/or specificity of the algorithm or to align with new evidence (Table 2).

All tools included additional or modified content to enhance sensitivity and/or specificity of the algorithm or to align with new evidence.

This was particularly notable for children presenting with fever. The IMCI chart booklet focuses fever assessment on malaria and measles. Beyond these 2 conditions, it only advises health care providers to “Look for any bacterial cause of fever [and]… Give appropriate antibiotic treatment for an identified bacterial cause.”^5^ The footnotes list several symptoms and signs, but no clear diagnostic or management criteria are provided. Participants noted, from experience and literature, that these broad recommendations often led health care providers to overprescribe antibiotics. For this reason, most tools provided decision support for the assessment and management of common or serious bacterial causes of fever, with most also including additional diagnostic tests.

The common rationale for extending or modifying content was to enhance quality of care and support better use of resources, particularly antimicrobials. The degree of modifications varied across the CDSS according to the type of intervention, stakeholder priorities, and developer and stakeholder perceptions of the capacity to support health care providers to appropriately implement more complex algorithms.

### Revised Structure

**FIGURE 1:**
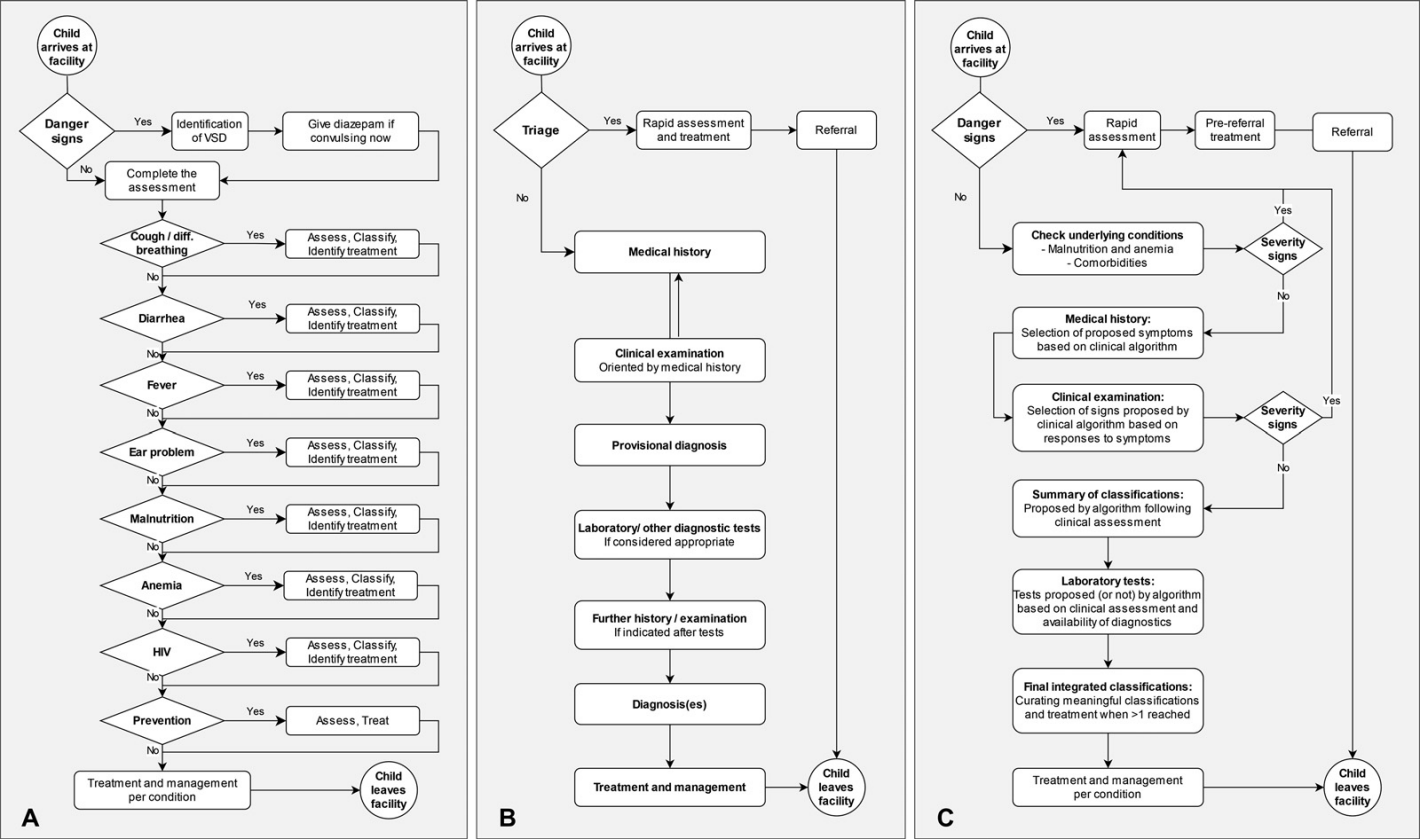
Consultation Workflows: A. Representation of the WHO IMCI Chart Booklet workflow^a,b^; B. Generic clinical consultation workflow^c^; C. Hybrid workflow combining elements of the symptom-driven workflow (A) With natural consultation process (B)^d^ Abbreviations: IMCI, Integrated Management of Childhood Illness; VSD, very severe disease; WHO, World Health Organization. ^a^ Integrated eDiagnosis Approach follows a similar workflow but also includes a registration/triage step in which temperature, anthropometric measurements, and malaria rapid diagnostic test (if fever) are conducted, with this information being entered later in the workflow. ^b^ Prevention: includes immunization, vitamin A, deworming, nutrition counseling, TB, HIV, etc. ^c^ Electronic Point-of-Care Test Plus follows a similar workflow. ^d^ Algorithm for the Management of Childhood Illnesses and MSFeCARE-Ped implemented workflows similar to this.

The IMCI chart booklet follows a linear process in which, for each presenting symptom or syndrome, the health worker is advised to “ask” certain questions, then “look, (listen), feel” for signs (and perform measurements/tests where relevant) to classify and identify treatment before moving on to the next presenting symptom/issue (Figure 1A). This modular syndrome-based assessment approach is not aligned with the usual flow of a clinical consultation, where similar tasks are grouped together (Figure 1B). Although the IMCI chart booklet presents syndromes in a set order, health care providers can flip back and forth between charts.

CDSSs tend to enforce a predefined navigation through the clinical algorithm, enforcing mandatory responses to clinical prompts to ensure a systematic and complete (and thus safe) consultation process.^23^^,^^25^^,^^29^^,^^33^ This is defined by the clinical algorithm and the constraints of the digital solution. Therefore, the pathway structure varies between different CDSSs, with the overall workflow following a structure similar to IMCI guidance (Figure 1A), a classical primary care clinical encounter (Figure 1B), or a specifically tailored workflow (Figure 1C). Regardless of the exact workflow, all organizations emphasized the importance of ensuring that the CDSS should neither disrupt nor delay the consultation. Relative to IMCI guidance, the CDSSs implemented 2 types of modifications aimed at enhancing the consultation structure (Box 2).

BOX 2 Clinical Decision Support System Modifications Designed to Enhance Consultation Structure

1. Modifications to expedite the identification and management of children with severe illness:
  - Reordered certain assessments to bring those most likely to be associated with severe illness first
  - Created predefined shortcuts (skip logic) when a severe illness is identified to avoid unnecessary tasks and expedite prereferral treatment and referral
2. Modifications to integrate components of the assessment, diagnosis, and management to improve efficiency and user experience:
  - Reordered certain assessments to bring those to the beginning that may influence other diagnoses (e.g., anemia, malnutrition) or to the end that rely on synthesizing information from other components of the consultation (e.g., fever without identified source)
  - Grouped similar tasks according to stages of the consultation (i.e., grouping together medical history items, examination items, diagnostic tests, diagnoses, and management rather than performing each according to presenting syndrome before moving to the next)
  - Integrated different diagnoses and treatments to ensure relevance of proposed final classifications and management recommendations

## COMPARISON OF PROCESSES FOR CDSS DEVELOPMENT

**FIGURE 2:**
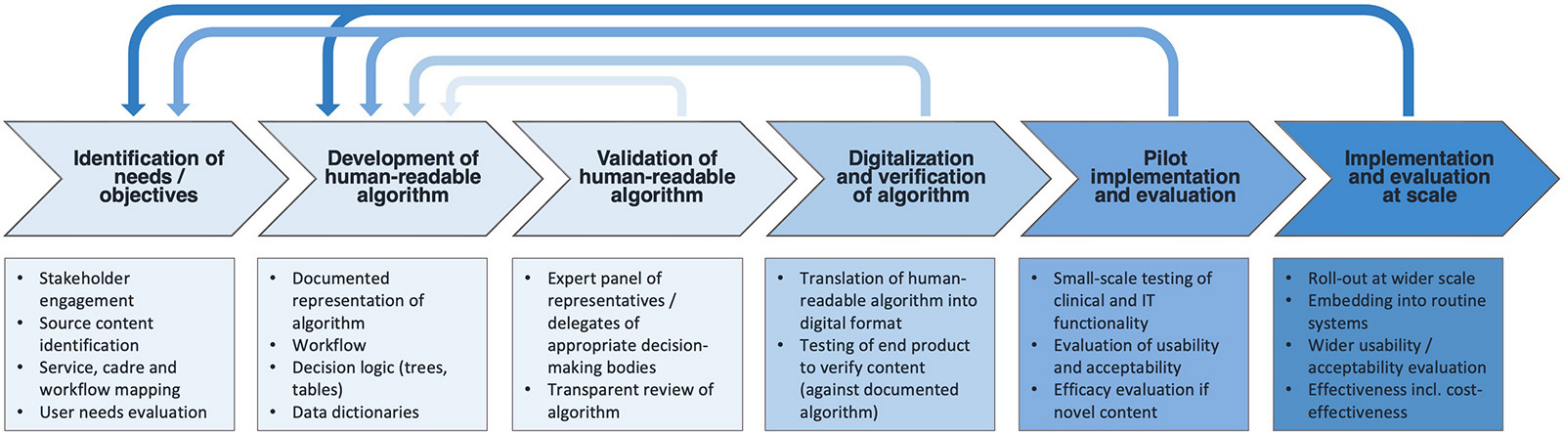
High-Level Overview of Processes Involved in the Development and Implementation of Clinical Decision Support Systems, With Feedback Loops Between Stages

The process of development and refinement of clinical algorithms can take several years, from the identification of needs and objectives of a CDSS through development to implementation and evaluation. Though various steps in the process can be outlined in a linear fashion, there are many feedback loops for iterations of the algorithm over time (Figure 2). We focus here solely on the clinical algorithm development process. Although the details of the process differed between each CDSS, several common steps were identified across organizations.

### Sources of Additional Content

All organizations needed to draw on several different sources in the process of algorithm development (Table 3). Alongside national (and/or generic WHO) IMCI guidelines, primary care health care providers are often expected to adhere to various other national or international clinical guidelines, including disease-specific guidelines (such as those for malaria, tuberculosis, or HIV), national formularies, or standard treatment guidelines. Most of the CDSSs integrated several different child health guidelines within their clinical algorithms.

**TABLE 3.** Sources or Reference Documents Used in the Adaptation and/or Development of New Content and Content-Validation Committee

**Source(s)**	**Rationale**	**CDSS**
IMCI guidelines	WHO generic and/or national IMCI guidelines to reflect country specific adaptations. May involve updates based on national/international guidelines (e.g., national pneumonia guidelines may have been updated more recently than IMCI).	All
Other national or international guidelines (e.g., standard treatment guidelines, formularies, disease-specific guidelines for malaria, TB, HIV, etc.)	To extend scope (relative to IMCI) by incorporating information from various guidelines relevant to the diagnosis and management of children in primary care (that health workers are expected to implement at a country level) or to reflect most up-to-date relevant guidance not yet incorporated into national guidelines due to long guideline update cycles (e.g., WHO young infant guidelines).	All
Existing or newly conducted evidence syntheses (systematic reviews/meta-analyses)	To implement relevant guidance not yet incorporated into national or international guidelines (due to long guideline update cycles), particularly if extending scope.	ALMANACH, MSFeCARE-Ped, ePOCT+
Epidemiological data (surveillance/research)	To target focus for additional scope/content (on conditions with significant morbidity or mortality). To incorporate flexibility within the algorithm (e.g., diagnostic and treatment advice differing based on location of facilities in high/low prevalence areas for diseases such as malaria).	MSFeCARE-Ped, ePOCT+, IeDA
Existing clinical algorithm	To use work already conducted on translating narrative guidance into decision logic, including updates that may have been made over time based on implementation experience or novel diagnostic/treatment approaches that may have been evaluated elsewhere.	All
Expert opinion (national or international panel)	To advise which source documents (guidelines or other evidence) should be used as a basis for the algorithm. To advise/review/validate interpretation of narrative guidelines/evidence translation to human-readable algorithm.	All

Abbreviations: ALMANACH, Algorithm for the Management of Childhood Illnesses; ePOCT+, Electronic Point-of-Care Test Plus; IeDA, Integrated eDiagnosis Approach; IMCI, Integrated Management of Childhood Illness; WHO, World Health Organization.

Due to lengthy update cycles, some guidelines may not reflect the latest evidence (and some guidelines may conflict with each other). Furthermore, many narrative clinical guidelines do not explicitly provide the decision logic necessary to develop clinical algorithms. Therefore, clinical algorithm developers also consulted peer-reviewed literature, other guidelines, and national or international expert opinion to ensure that algorithms reflected current evidence and national policymaker perspectives.

The extent to which different sources were drawn varied according to the CDSS purpose and context (Table 3), but all organizations agreed that early and ongoing engagement with stakeholders is needed to determine the relevant sources for the algorithm.

Finally, the algorithms themselves may become a source (i.e., once they have been developed for 1 country, the algorithm may provide a basis or framework for an algorithm in another country [with adaptation to national guidelines]).

### Human-Readable Format of the Clinical Algorithm

To enable experts’ review and validation of the clinical algorithm and ensure transparency of the content to relevant stakeholders, all participants agreed on the importance of a clearly documented, human-readable representation of the clinical algorithm.

In the absence of universally agreed standards for clinical algorithm representation at the time of their development, each organization addressed this issue slightly differently. All organizations developed diagrammatic representations, with some demonstrating the entire algorithm and others grouped according to either syndromes, diagnoses, or stages of the consultation. Some organizations used business process model and notation standards; others developed decision tables. Despite differences in format, the common rationale for these representations was to demonstrate the algorithms’ clinical workflow, content, and decision logic to support validation by experts and/or end users.

### Modifications and Updates

All organizations recognized that modifications are required following digitalization of the clinical algorithm during testing, piloting, and implementation. Given the complex nature of the algorithms, interaction with the end product often uncovers issues not apparent in the human-readable written (or depicted) format. Issues may be identified during verification (ensuring that the digital end product represents the human-readable algorithm) before it is in the hands of end users. Others are only identified from health worker feedback, observation, or analysis of CDSS or research data during piloting or implementation. Lastly, updates to narrative guidelines or new evidence may necessitate a need to update the clinical algorithm.

Participants acknowledged the importance of maintaining an up-to-date human-readable algorithm to ensure transparency of substantive updates to the digital algorithm (i.e., clinical content rather than user interface), which can be validated by relevant experts.

## DISCUSSION

This comparison reflects the collective experience of several organizations in clinical algorithm development for CDSSs targeting the diagnosis and management of childhood illness in primary care in resource-constrained settings. In identifying the commonalities and differences in scope, content, structure, and development processes, we aim to highlight important considerations in the development of clinical algorithms for this population and contribute to the global dialogue on improving transparency, trust, and quality of health worker decision support.

Rigorously and transparently developing CDSS clinical algorithms is a complex and lengthy process with many similarities to guideline adaptation.^43^ Context was acknowledged as an important critical driver of the content, structure, and development process of clinical algorithms for the CDSSs included in our analysis. Epidemiology, resources, clinical workflow, and the programmatic context (from controlled research settings to long-term health systems strengthening interventions) all influenced the degree to which the clinical algorithm deviated from IMCI guidance. Yet across all contexts, efficiency and fit to the consultation were recognized as critical in ensuring clinical safety and promoting uptake.

Context was acknowledged as an important critical driver of the content, structure, and development process of clinical algorithms for the CDSSs included in our analysis.

All algorithms drew on sources beyond IMCI guidance, including other national and international guidelines, published evidence, and expert opinion. Context was critical in determining the extent of additional sources used—from the availability of up-to-date guidelines to stakeholder priorities—and the extent of the expectation of health care providers to integrate many (sometimes conflicting) guidelines. Extending scope and content provides opportunities to improve quality of care, known to be worse for non-IMCI problems,^44^ and uptake by health care providers, who report challenges when not supported by a diagnosis,^39^ though further understanding on the usability and impact of more complex content is needed. Incorporating wider evidence was found to be limited by the dearth of literature on prognostic and diagnostic predictors in pediatric primary care in low- and middle-income countries.^45^ These issues highlight the critical importance of addressing evidence gaps and of timely updates of international guidelines and guidance on adaptation for resource-constrained settings,^43^ whether guidance is in paper or digital form.

Though all organizations acknowledged the importance of clearly documented human-readable algorithms for validation and transparency, different representation approaches were taken. Various methods have been proposed to represent CDSS clinical algorithms, but it is recognized that no single representation can adequately capture the complexity.^46^ WHO’s SMART Guideline digital adaptation kits—which “distill WHO guidelines and operational resources into a standardized format that can be more easily incorporated into digital tracking and decision support systems”—along with the forthcoming handbook for digitizing primary health care, are an important step in enhancing the validity, transparency, and accessibility of CDSSs.^19^^,^^21^^,^^22^ Their description of the process undertaken for the development of a digital antenatal care module reflects many issues applicable to CDSSs for the diagnosis and management of childhood illness.^47^^,^^48^ Authors agreed that when digital adaptation kits become available for child health in primary care, stakeholders should collaborate to support adaptation, implementation, and evaluation while continuing to foster innovation to support future improvements in CDSS quality and impact.

### Limitations

This article has several limitations. First, it reflects the work of a predominantly Swiss-based working group, and although the authors have collaborated with ministries of health, nongovernmental organizations, academic institutions, civil society organizations, health care providers, and caregivers, this article does not directly reflect their views. We have since worked to address this by working with the Geneva Digital Health Hub on the formation of a broader CDSS Community of Practice. Although in its infancy, this group already includes a wider membership from many countries with whom we are collaborating to develop a common working approach and objectives. Further, this work does not represent an exhaustive list of IMCI-related CDSSs; however, from our network and literature search, we are only aware of 3 other IMCI-related CDSSs in South Africa, Bangladesh, and Tanzania (1 of which is no longer in use), and no others have been highlighted in a recent systematic review.^12^ Lastly, we have not addressed other important considerations in CDSS development and implementation. These include, among others: CDSS evaluation—from performance to usability and acceptability to clinical and cost-effectiveness in controlled settings and at scale^49^; the algorithm adaptation requirements for different levels of care or differing epidemiology within a country; and wider implementation considerations such as training and mentorship, operational support, IT systems interoperability, and regulation. These issues all influence the content, structure, and development process of clinical algorithms—and their uptake and impact—but were beyond the scope of this article.

## CONCLUSION

The results of this comparison reflect the first step by this group of practitioners, scientists, and policy actors in embracing the Principles for Digital Development^50^ to collectively share learning and expertise on CDSSs for IMCI in primary care. Further evaluation of the relative effectiveness and cost-effectiveness of different approaches is needed to guide evidence-based practice in this complex field. Building and adhering to standards for CDSS development and implementation through multistakeholder dialogue is critical to ensure digital tools can effectively and equitably contribute to improve health and quality of care for children in resource-constrained settings.

## Notes

**Peer Reviewed**

**First published online:** July 27, 2023.
